# Author Correction: m^6^A modification of mutant huntingtin RNA promotes the biogenesis of pathogenic huntingtin transcripts

**DOI:** 10.1038/s44319-026-00870-w

**Published:** 2026-07-17

**Authors:** Anika Pupak, Rodríguez-Navarro Irene, Kirupa Sathasivam, Ankita Singh, Amelie Essmann, Daniel del Toro, Silvia Ginés, Ricardo Mouro Pinto, Gillian P Bates, Ulf Andersson Vang Ørom, Eulàlia Martí, Verónica Brito

**Affiliations:** 1 Departament de Biomedicina, Facultat de Medicina, Institut de Neurosciències, Universitat de Barcelona, Barcelona, Spain; 2 Institut d’Investigacions Biomèdiques August Pi i Sunyer (IDIBAPS), Barcelona, Spain; 3 Centro de Investigación Biomédica en Red sobre Enfermedades Neurodegenerativas (CIBERNED), Madrid, Spain; 4 Department of Neurodegenerative Disease, Huntington’s Disease Centre and UK Dementia Research Institute at UCL, Queen Square Institute of Neurology, UCL, London, WC1N 3BG UK; 5 Department for Molecular Biology and Genetics, Aarhus University, Aarhus C, Denmark; 6 Department of Biomedicine, Aarhus University, Aarhus C, Denmark; 7 Center for Genomic Medicine, Massachusetts General Hospital, Boston, MA USA; 8 Department of Neurology, Harvard Medical School, Boston, MA USA; 9 Program in Medical and Population Genetics, Broad Institute of MIT and Harvard, Cambridge, MA USA; 10 Centro de Investigación Biomédica en Red de Epidemiología y Salud Pública (CIBERESP), Madrid, Spain

**Correction to:** *EMBO Reports* (2024) 25:5026–5052. 10.1038/s44319-024-00283-7 | Published online 11 October 2024

The authors contacted the journal after identifying an omission in the Acknowledgements section of the published article.

**The Acknowledgements section is corrected**.

The Acknowledgements section is corrected from:

*We are very grateful to Ana Lopez and Maria Teresa Muñoz for technical assistance, Dr Teresa Rodrigo and the staff of the animal care facility (Facultat de Psicologia Universitat de Barcelona). We acknowledge Dr. Eva Novoa and Ana Milanovic from Center of Genomic Regulation for basecalling and analysis of Direct RNA sequencing datasets for the detection of m6A modifications in the Htt gene*.

*This work was supported by the Ministerio de Ciencia, Innovación y Universidades (PID2020-116474RB-100 to VB, PID2020-113953RB-I00 to EM and RTI2018-094374-B100 to SG); Hereditary Disease Foundation grant to VB; National Institutes of Health grant to RMP (R01 NS126420); PhD grant program by La Generalitat de Catalunya (2018FI_B_00487) to AP; PhD grant from PID2020-116474RB-100 (RE2021-097199) to IR*.

To: (Changes in bold)

*We are very grateful to Ana Lopez and Maria Teresa Muñoz for technical assistance, Dr Teresa Rodrigo and the staff of the animal care facility (Facultat de Psicologia Universitat de Barcelona). We acknowledge Dr. Eva Novoa and Ana Milanovic from Center of Genomic Regulation for basecalling and analysis of Direct RNA sequencing datasets for the detection of m6A modifications in the Htt gene* *.*

*This work was supported by* ***grant CNS2023-144738 funded by MICIU/AEI/10.13039/501100011033 and the European Union NextGenerationEU/PRTR to VB; by the**** Ministerio de Ciencia, Innovación y Universidades (PID2020-116474RB-100 to VB, PID2020-113953RB-I00 to EM and RTI2018-094374-B100 to SG); Hereditary Disease Foundation grant to VB; National Institutes of Health grant to RMP (R01 NS126420); PhD grant program by La Generalitat de Catalunya (2018FI_B_00487) to AP; PhD grant from PID2020-116474RB-100 (RE2021-097199) to IR*.

**Author statement:**

The authors confirm that the omission was inadvertent and apologize for the oversight. The correction does not affect the results, interpretations, or conclusions of the study.

