# Supplementary material for: m6A modification of mutant huntingtin RNA promotes the biogenesis of pathogenic huntingtin transcripts
Source: EMBO Rep. 2024 Oct 11;25(11):5026–52. doi: 10.1038/s44319-024-00283-7 (PMC11549361; doi:10.1038/s44319-024-00283-7)
Supplement: Supplementary file 1 — Appendix [file 44319_2024_283_MOESM1_ESM.pdf]

APPENDIX

Table of content:

- **Appendix Figure S1.** MERIP-qPCR approach enriches for m6A methylated transcripts……………………….Page 2
- **Appendix Figure S2.** m6A methylation levels of Htt1a transcripts are increased in *STHdh*$^{Q111/Q111}$ cells…....Page 3
- **Appendix Figure S3.** Correlation of m^{6}A methylation ratio with disease stage and CAG repeat length…...…Page 4
- **Appendix Figure S4.** *HTT1a* expression in human HD samples……………………………………………...….Page 4
- **Appendix Figure S5.** Pharmacological inhibition of METTL3 by STM2457 in ST*Hdh*$^{Q111/Q111}$ cells……..…Page 5 and ST*Hdh*$^{Q7/Q7}$ cells
- **Appendix Figure S6.** *Htt* RNA sequence of chimeric ST*Hdh*$^{Q111/Q111}$ cells/ *Hdh*$^{+/Q111}$ mice and potential DRACH motifs targeted by the CRISPRdCas13 approach……………………………………………………..…Page 6
- **Appendix Figure S7.** Fusion protein dCas13b-ALKBH5 is expressed in stable transfected ST*Hdh*$^{Q111/Q111}$ cells and only affect *Htt1a* expression……………………………………………………………...Page 7

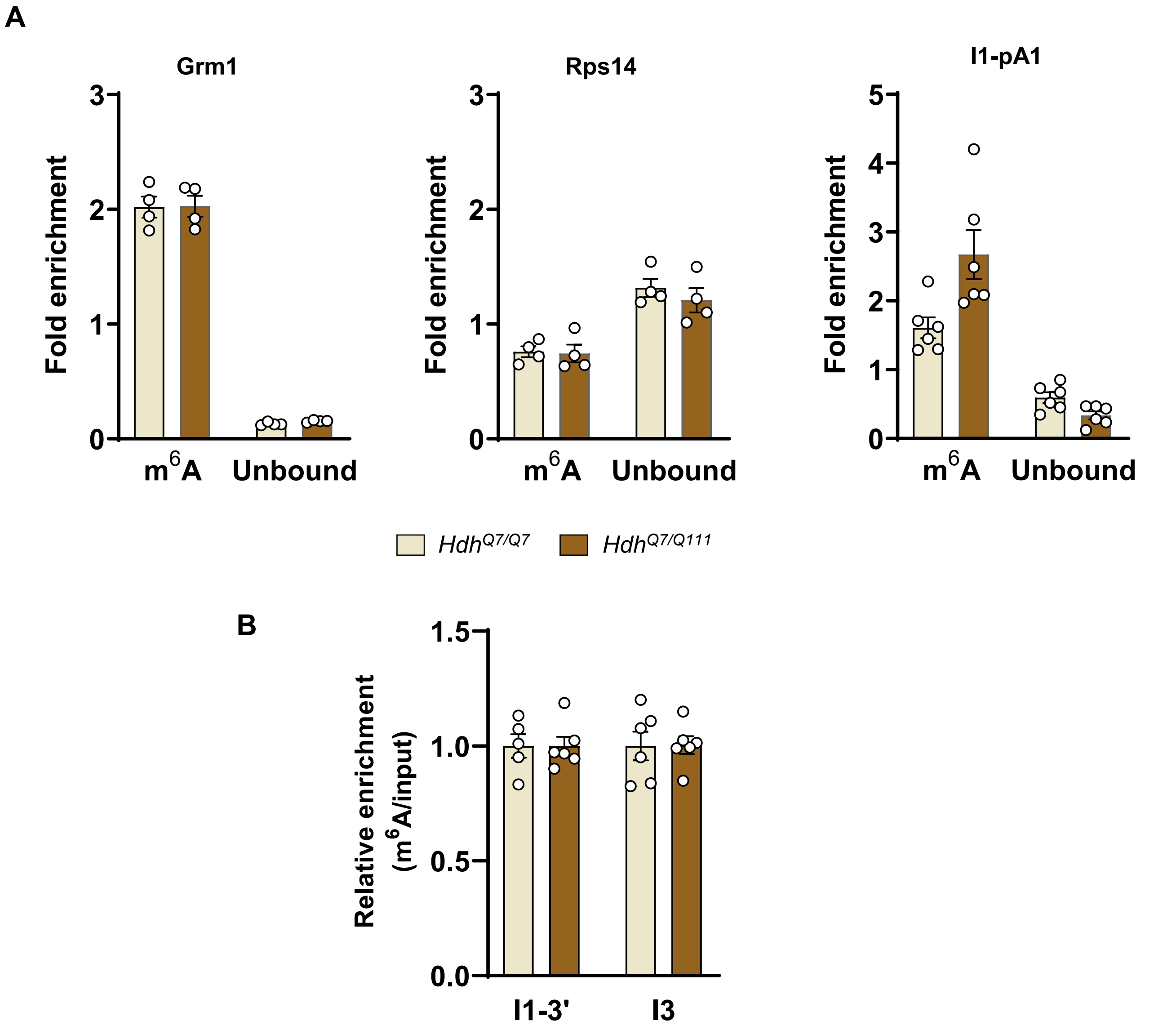

Appendix Figure S1. MERIP-qPCR approach enriches for m6A methylated transcripts. (A) m^{6}A enrichment (2^-(Ct of target gene in IP or unbound sample-Ct of target gene in input sample)) of positive (Grm1, n=4/genotype), negative (Rsp14, n=4/genotype) and I1-PA1 (n=6/genotype) transcripts obtained in the immunoprecipitated and unbound fractions of the MeRIP-qPCR of *Hdh*$^{Q7/Q7}$ and *Hdh*$^{Q7/Q111}$ mouse samples. **(B)** Relative m6A enrichment of I1-3 and I3 sequences in the immunoprecipitated samples of *Hdh*$^{Q7/Q7}$ and *Hdh*$^{Q7/Q111}$ mouse samples (n=5-6/genotype)

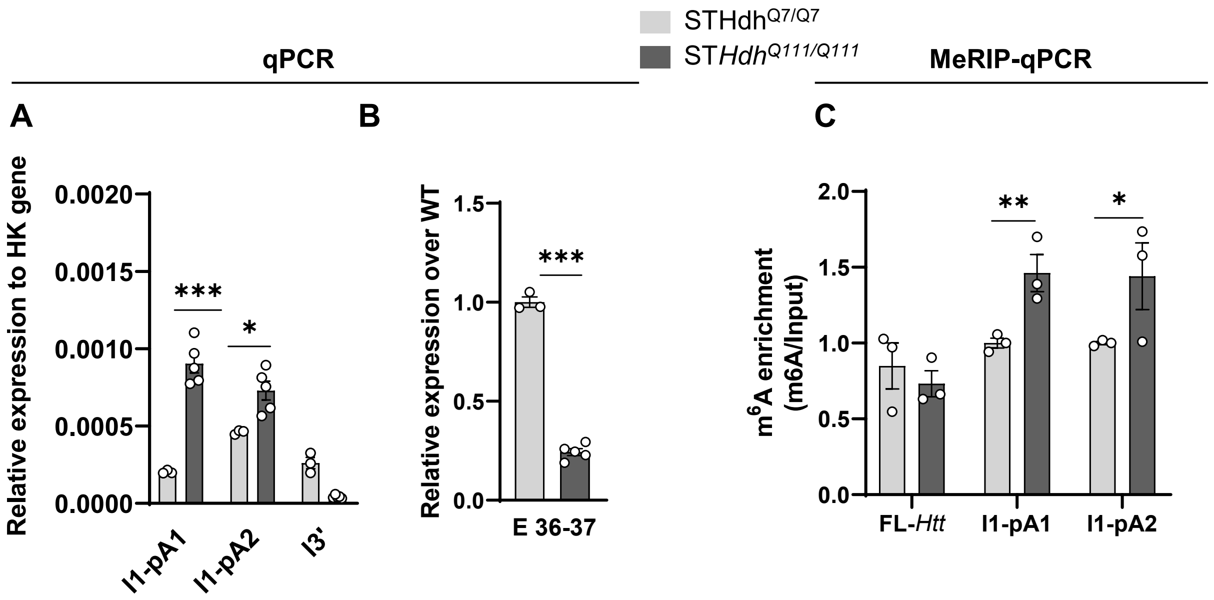

Appendix Figure S2. m⁶A methylation levels of *Htt1*a transcripts are increased in ST*Hdh*$^{Q111/Q111}$ cells. qPCR analysis of **(A)** intronic sequences and **(B)** FL-*Htt* expression levels in the ST*Hdh* immortalized striatal cells (n=3-5/ genotype)**.** Expression of intronic sequences is presented relative to housekeeping gene and FL-*Htt* levels are shown relative to WT **(C)** MeRIP-qPCR analysis in the ST*Hdh* immortalized striatal cells (n= 3/ genotype). Immunoprecipitated m6A transcripts are normalized to input and m6A enrichment is shown relative to WT. Data represent the mean ± SEM. Data were analyzed using Student´s two-tailed t-test. *$p < 0.05$, ***$p < 0.01$ and ***$p < 0.001$ compared with WT.

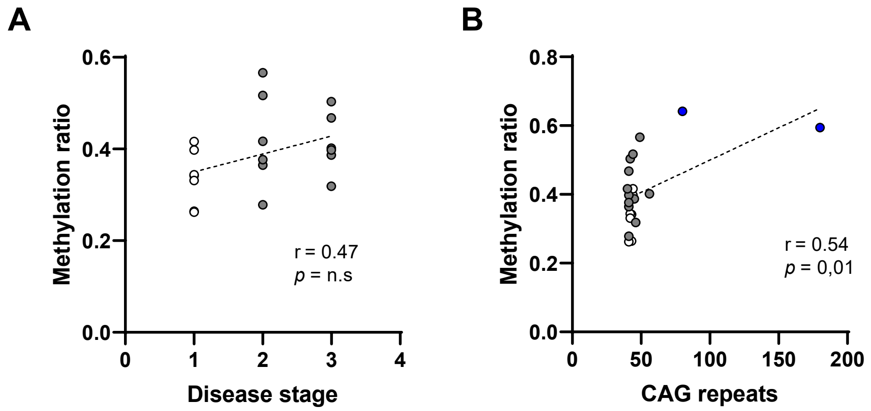

Appendix Figure S3. Correlation of m^{6}A methylation ratio with disease stage and CAG repeat length . Scatter plots showing correlation analysis between m6A levels at the GGACA motif and **(A)** different disease stages of HD patients skin fibroblasts; 1: presymptomatic; 2: symptomatic initial; 3: symptomatic moderate/advance **(B)** and CAG repeats length (from blood) of HD patients skin fibroblasts. Each dot represents an individual sample (white dots: Pre-HD; grey dots (S-HD), blue dots (HD juveniles). Correlation analyses in **A** were performed using Spearman's correlation test and in **B** using Pearson's correlation test. The correlation coefficient (r) and *p*-value are indicated in the plot. The solid line represents the best-fit linear regression line. Statistical significance was defined as $p < 0.05$

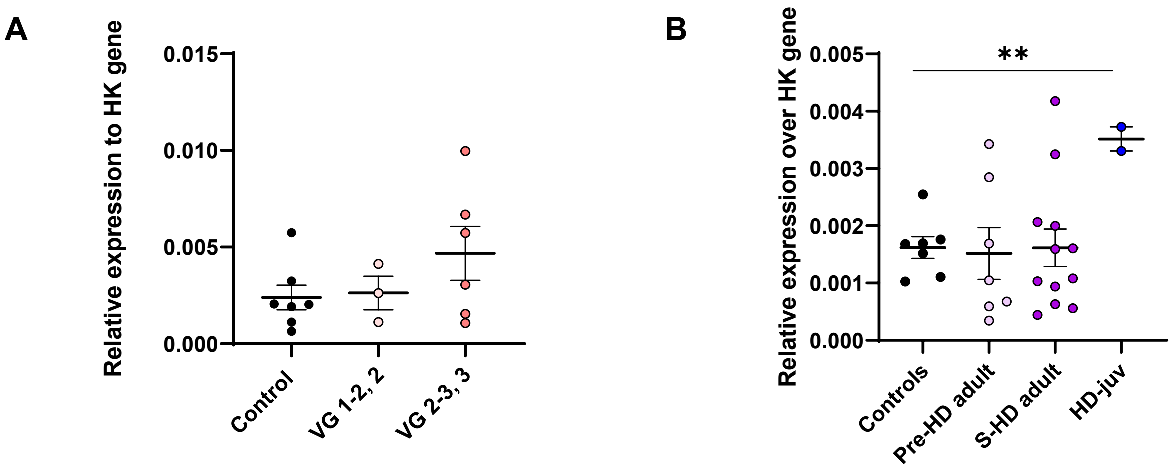

Appendix Figure S4. *HTT1a* expression in human HD samples. (A) qPCR analysis in putamen extracts of post-mortem brain tissue (n= 3-7 individuals/group) and **(B)** in human skin fibroblasts (n=2-12 individuals/group). VS: Vonsattel grade. Pre-HD adult: presymptomatic; S-HD adult: symptomatic**;** HD-juv: HD juvenile (Q80 and Q180). Data represent the mean ± SEM. Data were analyzed using Student t test. $^{**}p < 0.05$

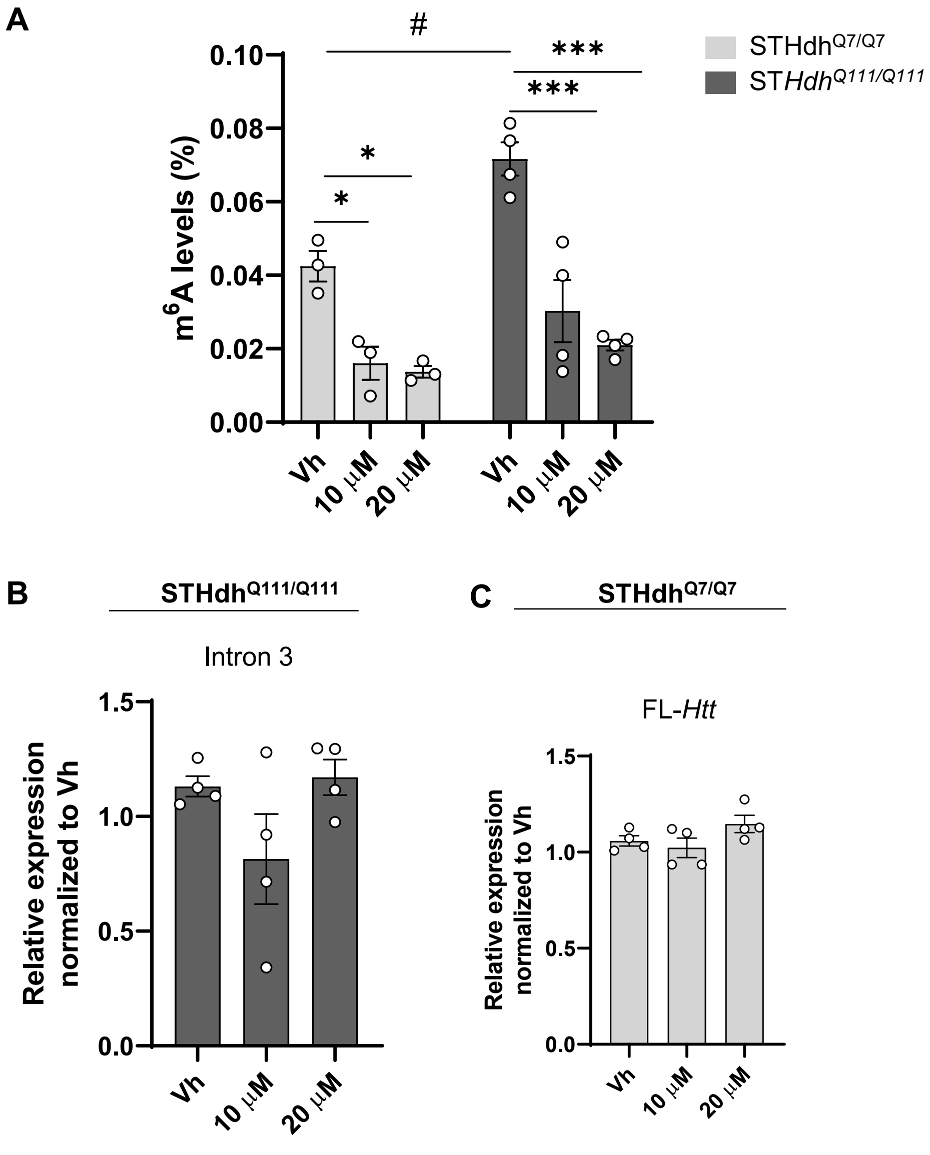

Appendix Figure S5. Pharmacological inhibition of METTL3 by STM2457 in ST*Hdh*$^{Q111/Q111}$ cells and ST*Hdh*$^{Q7/Q7}$ cells. (A) Overall m^{6}A levels were measured using EpiQuik m^{6}A RNA Methylation Quantification Kit in ST*Hdh*$^{Q7/Q7}$and ST*Hdh*$^{Q111/Q111}$ cells. Histograms show percentage of m^{6}A levels in total RNA (n= 3-4 independent experiments; 2 technical replicates/experiment). Data represent the mean ± SEM. Data were analyzed using One-way ANOVA with Tukey´s multiple comparisons test. *$p < 0.05$, ***$p < 0.001$ compared with cells treated with DMSO (Vh) and #$p < 0.01$ compared with ST*Hdh*$^{Q7/Q7}$ cells **(B)** qPCR analysis of the I3 *Htt* transcript in ST*Hdh*$^{Q111/Q111}$ cells treated with DMSO (Vh), STM2457 10 µM and STM2457 20 µM for 48h (n = 4 independent experiments). Data represent the mean ± SEM. Data were analyzed using One-way ANOVA with Tukey´s multiple comparisons test **(C)** qPCR analysis of FL-*Htt* in ST*Hdh*$^{Q7/Q7}$ cells treated with DMSO (Vh), STM2457 10 µM and STM2457 20 µM for 48h (n = 4 independent experiments). Data represent the mean ± SEM. Data were analyzed using One-way ANOVA with Tukey´s multiple comparisons test.

A

Sequence hm/ms Exon1-Intron 1 of *Htt RNA in* ST*Hdh*$^{Q111/Q111}$ *cells and Hdh*$^{+/Q111}$ *mice*

GCACTCGCCGCGAGGGTTGCCGGGACGGGCCCAAGATGGCTGAGCGCCTTGGTTCCGCTTCTGCCTGCCGCGCAGAGCCCCAT
TCATTGCCTTGCTGCTAAGTGGCGCCGCGTAGTGCCAGTAGGCTCCAAGTCTTCAGGGTCTGTCCCATCGGGCAGGAAGCCGTC
ATGGCAACCCTGGAAAAGCTGATGAAGGCTTGCGGCGCCGCGAGTCGGCCCGAGGCCTCCGGGGACTGCCGTGCCGGGCGGG
AGACCGCCATGGCGACCCTGGAAAAGCTGATGAAGGCCTTCGAGTCCCTCAAGTCCTTCCAGCAGCAGCAGCAGCAGCAGCAG
CAGCAGCAGCAGCAGCAGCAGCAGCAGCAGCAGCAGCAGCAGCAGCAGCAGCAGCAGCAGCAGCAGCAGCAGCAGCAGCAGCAG
CAGCAGCAGCAGCAGCAGCAGCAGCAGCAGCAGCAGCAGCAGCAGCAGCAGCAGCAGCAGCAGCAGCAGCAGCAGCAGCAGCAG
CAGCAGCAGCAGCAGCAGCAGCAGCAGCAGCAGCAGCAGCAGCAACAGCCGCCACCGCCGCCGCCCCGCCGCCGCCTCCTCA
GCTTCCTCAGCCGCCGCCGCAGGCACAGCCGCTGCTGCCTCAGCCGCAGCCGCCCCCGCCGCCGCCCCCGCCGCCACCCGGCCC
GGCTGTGGCTGAGGAGCCGCTGCACCGACCgtgagtttgggcccgctgcagctccctgtcccggcgggtcccaggctacggcggggatggcggtaaccc
tgcagcctgcgggccggcgacacgaaccccggccccgcagagacagagtgacccagcaacccagagcccatgagggacacccgcccccctcctggggcgaggcctt
cccccacttcagccccgctccctcacttgggtcttcccttgtcctctcgcgaggggaggcagagccttgttggggcctgtcctgaattcgatggccctcgtcttgcggggtc
tctggcctccctcagaggagacagagccgggtcaggccagccagggactcgctgaggggcgtcacgactccagtgccttcgccgttcccagtttgcgaagttagggaa
cgaacttgtttctctcttctggagaaactggggcggtggcgcacatgactgttgtgaagagaacttggagaggcagagatctctagggttacctcctcatcaggcctaag
agctgggagtgcaggacagcgtgagagatgtgcgggtagtggatgacataatgcttttaggaggtctcggcgggagtgctgagggcgggggagtgtgaacgcatcca
atgggatattctttttccaagtgacacttgaagcagcctgtgactcgaggcacttcgtactctcctggcgtttcatttagtttgtggtgtagtgtagttaaaccaggttttaa
gcatagccagagaggtgtgcttctgtgtgtctgcaggcagttggatgagttgtatttgtcaagtacatggtgagttacttaggtgtgattattaataaaaaactatatgtgt
gcatatatatgaaagagtcgacttatacttaactgcctatcgatttttttgttctatataaaacggatacattggtggtgctcagttttcaccggggaatgaattttactagtg
ttgcagacaggcttgttttagaacataggccactctgactctgactttgtgccagtaaaagttcctgtttagttctttgctgacatcttatagatctttggaagctagctgctt
gtgactggagagaatattgaaacagaagagagaccatgagtcacagtgctctaagagaaaagacgctcaaaacatttcctggaaatccatgctgagtgttgagccctg
tgctctcttgcagctcagtcctttctctcaactctgggcattttatttctaatctggatttgtataattaataaggagaacttttgggaacaacctactaaagaatgtcatcat
taaaactcacttagaaaataagt

-Human insert sequence
-Mouse sequence
- m6A motifs
- m6A ACA sites analyzed by MazF-qPCR
- cryptic polyA1 site according Sathasivam *et al* [5]
- cryptic polyA2 site according Neueder *et al* [4]
- gRNA3
- gRNA2
- gRNA1

B **Targeted m6A sites analzyed by MazF-qPCR of the gRNAs used in CRISPR approach**

gRNA1	Upstream	GGACA hm: 560 nt<br>GGACAhum: 399 nt<br>GGACAms: 186 nt
	Downstream	-----
gRNA2	Upstream	GGACAhum: 207 nt<br>GGACAms: 46 nt
	Downstream	GGACAms: 167 nt
gRNA3	Upstream	GGACAhm: 90 nt
	Downstream	AGACAms: 70 nt<br>GGACAms: 284 nt

Appendix Figure S6. *Htt* RNA sequence of chimeric ST*Hdh*$^{Q111/Q111}$ cells/ *Hdh*$^{+/Q111}$ mice and potential DRACH motifs targeted by the CRISPRdCas13 approach. (A) Sequence of mutant *Htt* RNA (5' → 3') of ST*Hdh*$^{Q111/Q111}$ cells/ *Hdh*$^{+/Q111}$ mice. Sequence of the human insert is shown in orange and murine sequence in black. The indicated colors in the figure highlight m6A motifs in the first 524bp of intron1; the m6A-ACA sites analyzed by MazF qPCR; cryptic sites and gRNAs used for targeting. **(B)** Table showing the location of the target m6A sites analyzed by MazF qPCR for each gRNA used in the CRISPR approach.

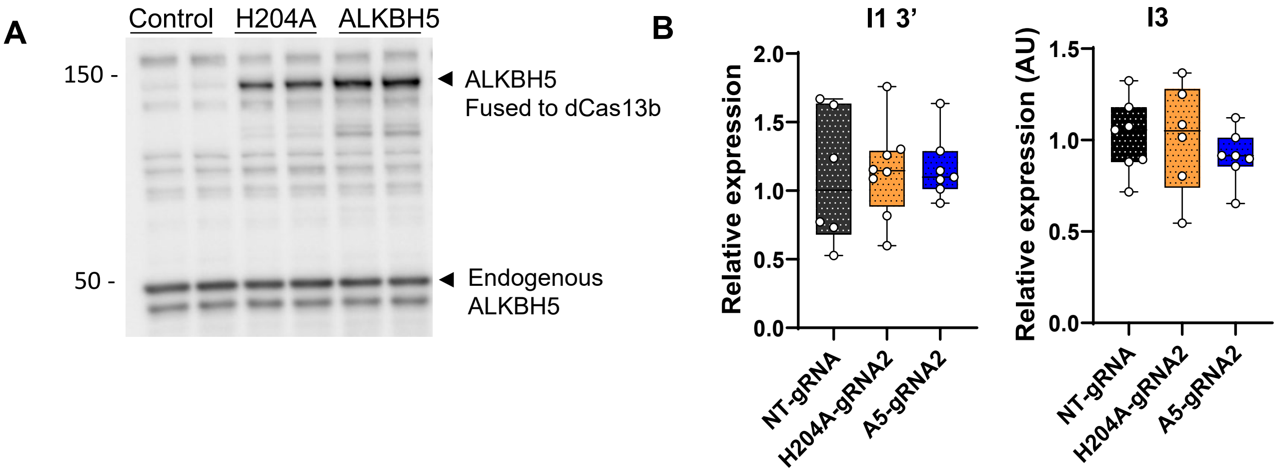

Appendix Figure S7. Fusion protein dCas13b-ALKBH5 is expressed in stable transfected ST*Hdh*$^{Q111/Q111}$ cells and only affect *Htt1a* expression (A) Representative Western Blots showing expression of fusion protein dCas13b-ALKBH5 and endogenous ALKBH5 (A5) in cells stably transfected with dCas13b-A5 NT-gRNA (control), dCas13b-H204 gRNA2 and dCas13b-A5 gRNA2. **(B)** Expression levels of I1-3’ and I3 *Htt* transcripts in transfected ST*Hdh*$^{Q111/Q111}$ cells with dCas13b NT gRNA, dCas13b-H204 gRNA2 and dCas13b-A5 gRNA2. (n=6-7 replicates). Data represent the mean ± SEM. Data were analyzed using One-way ANOVA with Tukey´s multiple comparisons test
