# Supplementary material for: m6A modification of mutant huntingtin RNA promotes the biogenesis of pathogenic huntingtin transcripts
Source: EMBO Rep. 2024 Oct 11;25(11):5026–52. doi: 10.1038/s44319-024-00283-7 (PMC11549361; doi:10.1038/s44319-024-00283-7)
Supplement: Supplementary file 2 — Table EV1 [file 44319_2024_283_MOESM2_ESM.pdf]

Table EV1. Details of human *postmortem* brain tissue. N/A: not available.

Identification	Diagnosis	Postmortem delay (h)	Vonsattel grade	CAG repeats
CS 1468	Control	0:00		
CS 1818	Control	5:00		
CS 1858	Control	7:30		
CS 1870	Control	7:20		
CS 1888	Control	5:30		
CS 1557	Control	7:25		
CS 2055	Control	15:45		
CS 1334	HD	7:00	1-2	40 (± 2)
CS 1980	HD	13:10	3	N/A
CS 1844	HD	15:30	2	42
CS 1933	HD	12:20	2	40
CS 1875	HD	8:00	2-3	39
CS 2060	HD	7:30	2-3	N/A
CS 1981	HD	12:30	3	N/A
CS 1630	HD	5:30	3	41
CS 1973	HD	6:30	3	N/A
