# Supplementary material for: m6A modification of mutant huntingtin RNA promotes the biogenesis of pathogenic huntingtin transcripts
Source: EMBO Rep. 2024 Oct 11;25(11):5026–52. doi: 10.1038/s44319-024-00283-7 (PMC11549361; doi:10.1038/s44319-024-00283-7)
Supplement: Supplementary file 3 — Table EV2 [file 44319_2024_283_MOESM3_ESM.pdf]

Table EV2. Details of human fibroblast lines. Stage of the disease (1: presymptomatic, 2: initial, 3: moderate-advanced).

Identification	Diagnosis	Age of onset	CAG repeats
**1**	Control		
**2**	Control		
**3**	Control		
**4**	Control		
**5**	Control		
**6**	Control		
**7**	Control		
**8**	Presymptomatic, stage 1		44
**9**	Presymptomatic, stage 1		44
**10**	Presymptomatic, stage 1		43
**11**	Presymptomatic, stage 1		43
**12**	Presymptomatic, stage 1		42
**13**	Presymptomatic, stage 1		41
**14**	Presymptomatic, stage 1		42
**15**	HD, stage 2	36	49
**16**	HD, stage 2	38	41
**17**	HD, stage 2	65	40
**18**	HD, stage 2	55	41
**19**	HD, stage 2	38	41
**20**	HD, stage 2	30	44
**21**	HD, stage 2	34	45
**22**	HD, stage 3	20	56
**23**	HD, stage 3	50	46
**24**	HD, stage 3	35	42
**25**	HD, stage 3	42	41
**26**	HD, stage 3	50	41
