# Supplementary material for: m6A modification of mutant huntingtin RNA promotes the biogenesis of pathogenic huntingtin transcripts
Source: EMBO Rep. 2024 Oct 11;25(11):5026–52. doi: 10.1038/s44319-024-00283-7 (PMC11549361; doi:10.1038/s44319-024-00283-7)
Supplement: Supplementary file 4 — Table EV3 [file 44319_2024_283_MOESM4_ESM.pdf]

Table EV3. List of siRNA for METTL3 knock-down

Identification	Company	Ref	Sequence
**siRNA_1*METTL3***	Ambion	AM16708	Sense: GGUCUCCUCCAAGAUGAUGtt
		ID:74203	Antisense: CAUCAUCUUGGAGGAGGAGACCtc
**siRNA_2*METTL3***	Ambion	AM16708	Sense: GGAGCCGGCUAAGAAGUCAtt
		ID185495	Antisense: UGACUUCUUAGCCGGCUCCtt
**siRNA*NTC***	Ambion	AM4611	Unknown
**siRNA_P*METTL3***	Satna Cruz	sc-149387	Unknown
