# Supplementary material for: m6A modification of mutant huntingtin RNA promotes the biogenesis of pathogenic huntingtin transcripts
Source: EMBO Rep. 2024 Oct 11;25(11):5026–52. doi: 10.1038/s44319-024-00283-7 (PMC11549361; doi:10.1038/s44319-024-00283-7)
Supplement: Supplementary file 5 — Table EV4 [file 44319_2024_283_MOESM5_ESM.pdf]

Table EV4. qPCR primer/probe sets used for gene expression analysis in mouse samples. For each assay, the sequences of the forward (FW) and reverse (RV) primers as well as the probe are provided, in addition to amplicon length and the IDT assay reference when purchased predesigned.

Assay name	ID	Sequence (5'→3')	Amplicon
***Htt* FL (Mm.PT.58.12088552)**	FW	CCGTTTCCACTTGTCCTCAT	93 bp
	RV	CTGTTACGACTCATCCAGTACC	
	Probe	AGATGTTCATCCTTGTCCTGCAGCA	
***Htt* PolyA 1/ MazF no ACA control**	FW	GGCGTCACGACTCCAGTG	84 bp
	RV	CCCCAGTTTCTCCAGAAGAGAGA	
	Probe	CCCAGTTTGCGAAGTTAGGGAACGA	
***Htt* PolyA 2**	FW	TTCCTGGAAATCCATGCTGAG	75 bp
	RV	AATGCCCAGAGTTGAGAGAAAG	
	Probe	CCTGTGCTCTCTTGCAGCTCAGTC	
**MazF GGACA mouse motif *Htt***	FW	TGAAGAGAACTTGGAGAGGCAG	99 bp
	RV	TCCACTACCCGCACATCTCT	
	Probe	AGGGTTACCTCCTCATCAGGCCTAA	
**MazF AGACA mouse motif *Htt***	FW	CTCCCTCAGAGGAGACAGAG	101 bp
	RV	TCCCTAACTTCGCAAACTGG	
	Probe	ACTCCAGTGCCTTCGCCGTT	
**MazF GGACA human motif *Htt***	FW	AGAGCCCATGAGGGACA	136 bp
	RV	TCGGTGAATTCAGGACAGG	
	Probe	CCTCACTTGGGTCTTCCCTTGTCC	
***Htt* Intron 1 3' (Obtained from Papadopoulou et al 2019)**	RV	TCCCTAACTTCGCAAACTGG	203 bp
	Probe	ACTCCAGTGCCTTCGCCGTT	
	Probe	CCCAAAGGTGCTAGCCTCCA	
***Htt* Intron 3**	FW	CAGGAAAGACCCTCCCATTT	99 bp
	RV	ACCCTCAAGACACACCATTT	
	Probe	ACCTGGAGGTTTGTCTCAGAGAGGA	
**Actinß (Mm.PT.39a.22214843.g)**	FW	GACTCATCGTACTCCTGCTTG	147 bp
	RV	GATTACTGCTCTGGCTCCTAG	
	Probe	CTGGCCTCACTGTCCACCTTCC	
***Grm1* (Mm.PT.58.31626536)**	FW	CTATGTCTCTGCAGTCCACAC	116 bp
	RV	TGCTGTAGATTTTGTCCGAGTG	
	Probe	AAGCATCCATTCCACTCTCGCCG	
**Rps14 (Mm.PT.58.11281550)**	FW	CCTTCCGCCTCTCTCTTTC	90 bp
	RV	CCTGCTCTTCCTTCTTTTCCT	
	Probe	CGCCATTTCTGATCGTCGTCTCCAG	
