# Supplementary material for: m6A modification of mutant huntingtin RNA promotes the biogenesis of pathogenic huntingtin transcripts
Source: EMBO Rep. 2024 Oct 11;25(11):5026–52. doi: 10.1038/s44319-024-00283-7 (PMC11549361; doi:10.1038/s44319-024-00283-7)
Supplement: Supplementary file 6 — Table EV5 [file 44319_2024_283_MOESM6_ESM.pdf]

Table EV5. qPCR primer/probe sets used for gene expression in human samples. For each assay, the sequences of the forward (FW) and reverse (RV) primers as well as the probe are provided, in addition to amplicon length and the IDT assay reference when purchased predesigned.

Assay name	ID	Sequence (5'→3')	Amplicon
**MazF no ACA control *HTT***	FW	GCCTTTCTCTTACCTCTCAGTATTC	144 bp
	RV	TGAAGACGAGACAAGGATCATAAC	
	Probe	TCTGGATGTGTCCCAGATGGCATT	
**MazF GGACA motif *HTT***	FW	AACCCAGAGCCCATGAG	149 bp
	RV	ACTCCCCTCGGTGAATTC	
	Probe	TCTTCCCTTGTCCTCTCGCGAGG	
**MazF AGACA motif *HTT***	FW	CTGATGGGAAGGAAGACCTTT	81 bp
	RV	CAGCATAAGAAAGAGAAGGCATATT	
	Probe	TGGGCTGCTTCAGACACTTGATCA	
**MazF TGACA motif *HTT***	FW	TCTCAGATGTGCCAGTGAAAG	94 bp
	RV	CCCAAGAACAAGCAACAGAAAC	
	Probe	CTGTTTGCTTCATTGCTGACAGCTTGT	
