# Supplementary material for: m6A modification of mutant huntingtin RNA promotes the biogenesis of pathogenic huntingtin transcripts
Source: EMBO Rep. 2024 Oct 11;25(11):5026–52. doi: 10.1038/s44319-024-00283-7 (PMC11549361; doi:10.1038/s44319-024-00283-7)
Supplement: Supplementary file 7 — Table EV6 [file 44319_2024_283_MOESM7_ESM.pdf]

Table EV 6. Primers used for PCR amplification of the CAG expansion region. For each assay, the sequence and the source are provided.

Assay name	Sequence (5'→3')	Amplicon
CAG1	ATCAAGGCCTTCGAGTCCCTCAAGTCCTCC	Mutant Q111~400 bp
HU3	GGCGGCTGAGGAAGCTGAGGA	
HTT_e1_Fw	ATGGCGACCCTGGAAAAG	60 bp
HTT_e1_Rev	CTGCTGCTGGAAGGACTTG	
