# Supplementary material for: m6A modification of mutant huntingtin RNA promotes the biogenesis of pathogenic huntingtin transcripts
Source: EMBO Rep. 2024 Oct 11;25(11):5026–52. doi: 10.1038/s44319-024-00283-7 (PMC11549361; doi:10.1038/s44319-024-00283-7)
Supplement: Supplementary file 8 — Table EV7 [file 44319_2024_283_MOESM8_ESM.pdf]

Table EV7. Primers used for 3'RACE. For each assay, the sequence and the source are provided. Primers not provided by the kit were purchased from IDT.

Assay name	Sequence (5'→3')	Source
GSP-pA1	AACCAGGTTTTAAGCATAGCCAGA	[5]
GSP-pA2	GCTGCTTGTGACTGGAGAGA	[4]
