# Supplementary material for: m6A modification of mutant huntingtin RNA promotes the biogenesis of pathogenic huntingtin transcripts
Source: EMBO Rep. 2024 Oct 11;25(11):5026–52. doi: 10.1038/s44319-024-00283-7 (PMC11549361; doi:10.1038/s44319-024-00283-7)
Supplement: Supplementary file 17 — Expanded View Figures [file 44319_2024_283_MOESM17_ESM.pdf]

Expanded View Figures

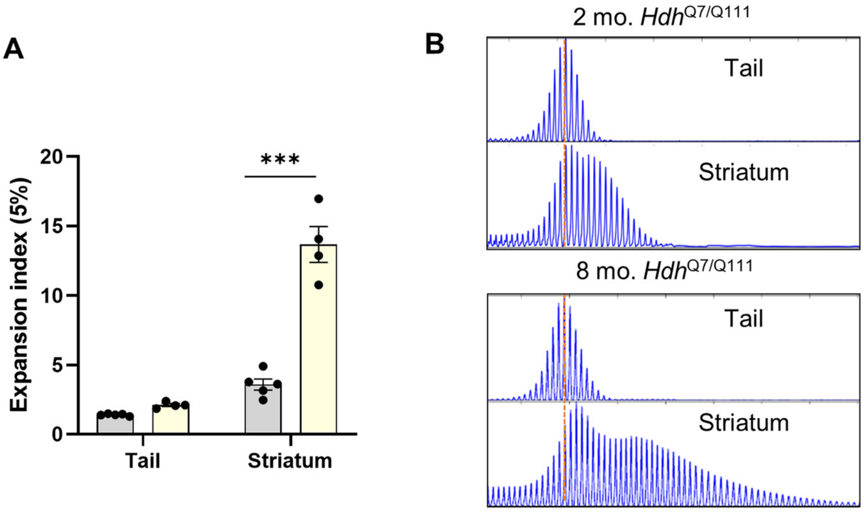

Figure EV1. Comparison of somatic CAG repeat instability in 2 and 8 months old *Hdh*$^{+/Q111}$ mice.

(**A**) Quantification of somatic expansion indices, of *Htt* CAG PCR products from tails and striatum of *Hdh*$^{+/Q111}$ at 2 and 8 months of age using a 5% peak height threshold. Error bars represent mean ± SEM; $n = 4$–5/age. Data were analyzed for each tissue using Student-T test, ***$P < 0.0001$. (**B**) Representative GeneMapper traces showing somatic CAG repeat expansions in the striatum and tails at the different ages analyzed. Source data are available online for this figure.

 © The Author(s)

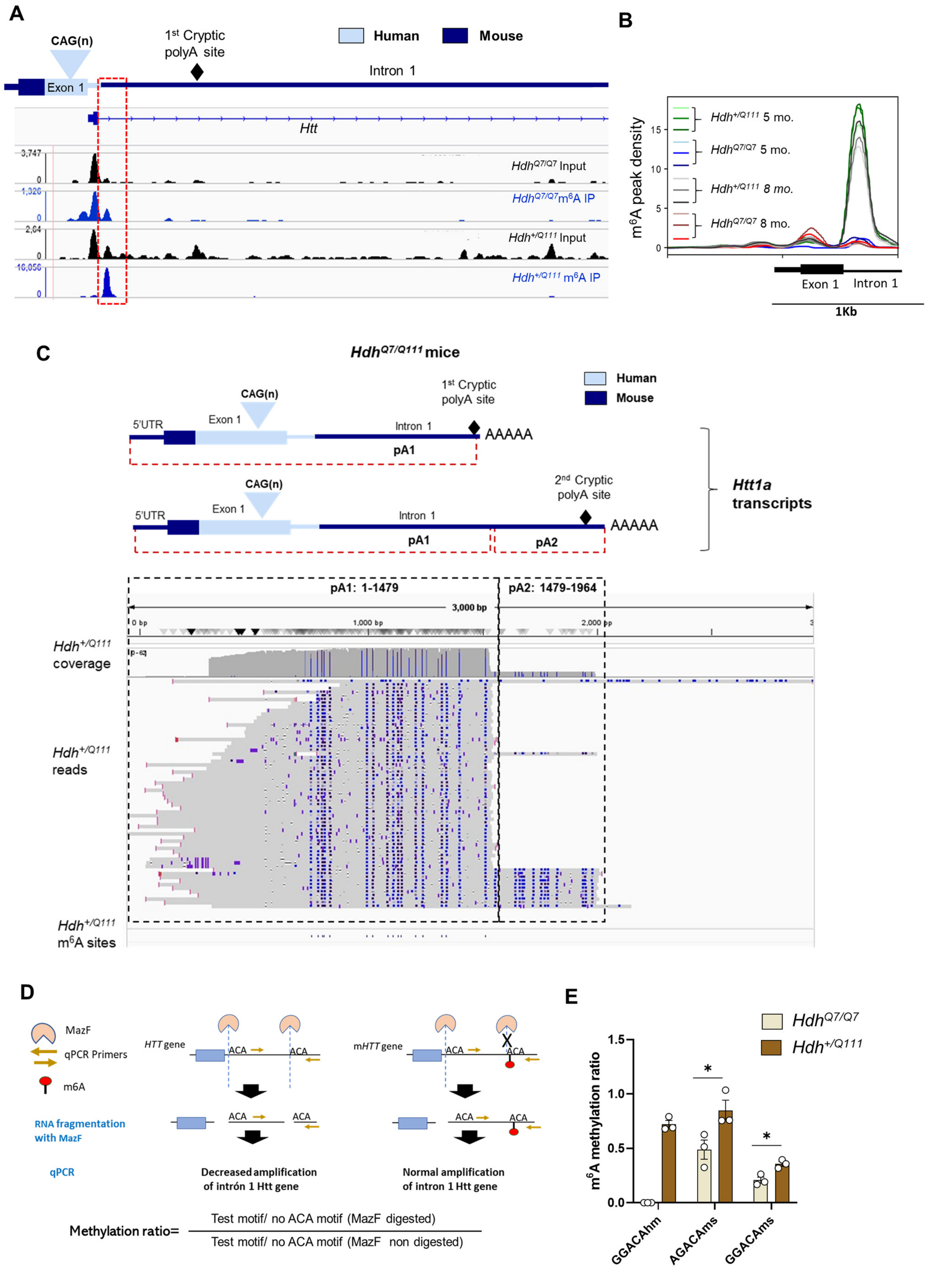
A
CAG(n)
1st Cryptic polyA site
Human
Mouse
Intron 1
Exon 1
Htt
HdhQ7/Q7 Input
HdhQ7/Q7 m6A IP
Hdh+/Q111 Input
Hdh+/Q111 m6A IP
B
m6A peak density
Hdh+/Q111 5 mo.
HdhQ7/Q7 5 mo.
Hdh+/Q111 8 mo.
HdhQ7/Q7 8 mo.
Exon 1
Intron 1
1Kb
C
HdhQ7/Q111 mice
5'UTR
Exon 1
CAG(n)
Intron 1
1st Cryptic polyA site
AAAAA
pA1
2nd Cryptic polyA site
pA2
Htt1a transcripts
pA1: 1-1479
pA2: 1479-1964
3,000 bp
Hdh+/Q111 coverage
Hdh+/Q111 reads
Hdh+/Q111 m6A sites
D
MazF
qPCR Primers
m6A
HTT gene
mHTT gene
ACA
RNA fragmentation with MazF
qPCR
Decreased amplification of intrón 1 Htt gene
Normal amplification of intron 1 Htt gene
Methylation ratio= Test motif/ no ACA motif (MazF digested) / Test motif/ no ACA motif (MazF non digested)
E
HdhQ7/Q7
Hdh+/Q111
m6A methylation ratio
GGACAhm
AGACAms
GGACAms

Figure EV2. m^{6}A enrichment in *Htt* intron 1 of *Hdh*$^{+/Q111}$ mice.

(**A**) Genome browser snapshots harboring m^{6}A enrichment in the proximal region of *Htt* intron 1 to the 5′ exon1-intron 1 splice site. The sequence data, narrow Peak, and alignment data supporting the data is available NCBI GEO repository under the accession code GSE175618 (Pupak et al, 2022). (**B**) Comparison of fold enrichment distribution of methylation sites in the *Htt* intron 1 between 8- and 5-month old WT and *Hdh*$^{+/Q111}$ mice obtained by MeRIP-seq ($n = 3$/genotype/age, Pupak et al 2022). (**C**) Mapping of m^{6}A sites in mutant *Htt1a* transcripts by direct RNA sequencing. IGV snapshot show m^{6}A sites in *Htt1a* transcripts in the striatum of *Hdh*$^{+/Q111}$ mice. Mouse sequence (GRCmm39 genome) of the *Htt* gene including the human insert was used as reference gene (chr5: 34,919,088–35,070,342). Purple and blue dots represent high and low confidence m^{6}A sites, respectively. (**D**) Schematic representation of the MazF-qPCR approach used for quantification of residue specific m^{6}A methylation. MazF interfase enzyme only cuts at the ACA sequence when not methylated allowing for interrogation of specific m^{6}A motifs and measurement of m^{6}A ratio following formula shown in the figure. (**E**) Methylation ratio of three different m^{6}A motifs obtained by MazF-qPCR analysis in the striatum of *Hdh*$^{Q7/Q7}$ and *Hdh*$^{+/Q111}$ ($n = 3$ mice/genotype). Data represent the mean ± SEM. Data were analyzed using Student-T test. *$P = 0.0416$ (AGACAms), *$P = 0.0144$ (GGACAms). Source data are available online for this figure.

 © The Author(s)

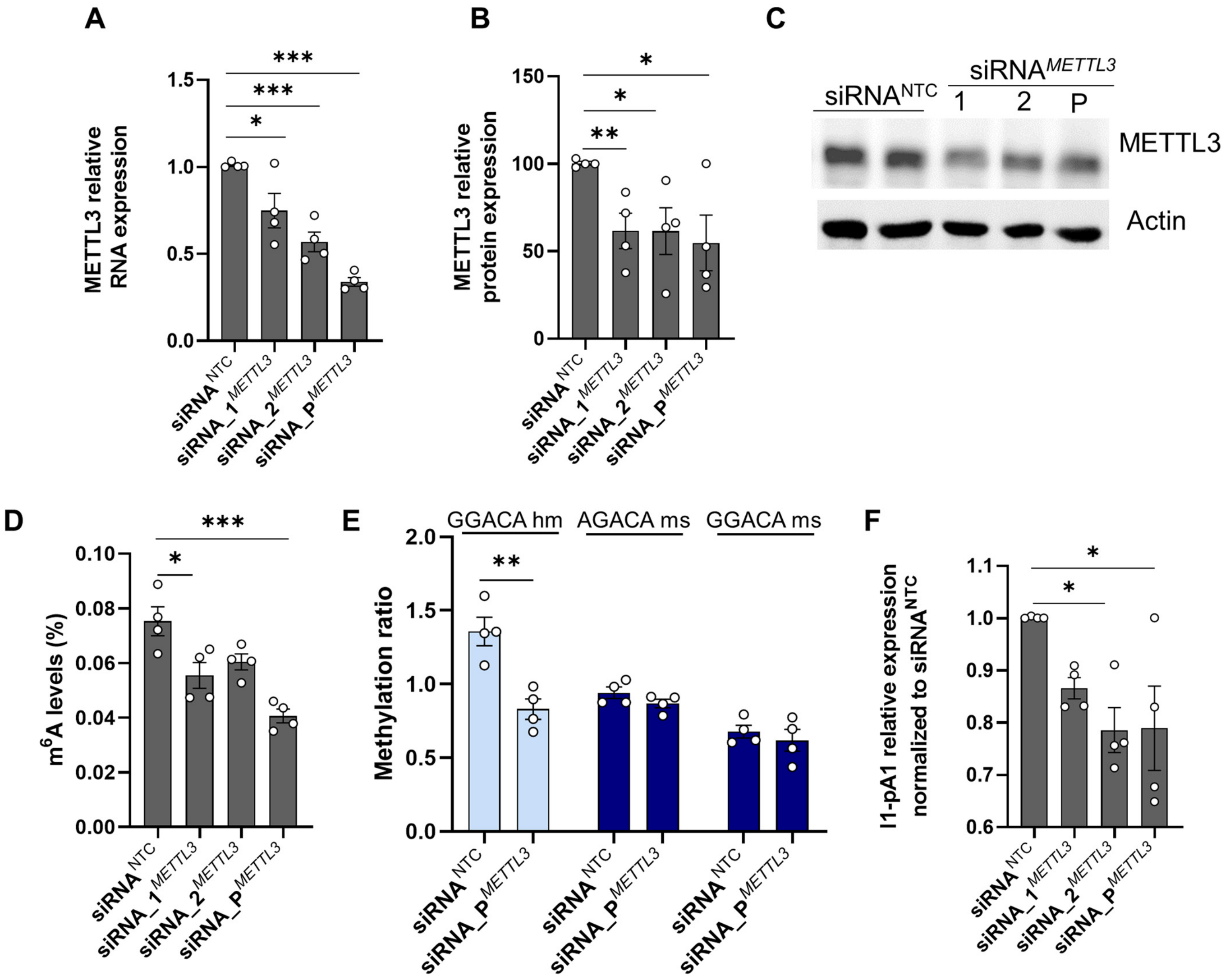

Figure EV3. METTL3 knockdown with siRNA decreases I1-pA1 levels in ST*Hdh*$^{Q111/Q111}$ cells.

METTL3 mRNA levels and protein expression levels analyzed by qPCR (**A**) and western blot (**B**) in ST*Hdh*$^{Q111/Q111}$ transfected for 24h with non-targeting control (siRNA^{NTC}), two different targeting sequences (siRNA_1^{METTL3} and siRNA_2^{METTL3}) and a pool of 3 target-specific siRNA (siRNA_P^{METTL3}) against *METTL3*. (**A**) qPCR analysis of the expression levels of *METTL3* ($n = 3$–4 independent experiments; 2 technical replicates/experiment). Data represent the mean ± SEM. Data were analyzed using one-way ANOVA with Tukey´s multiple comparisons test. *P =0.0358 (siRNA^{NTC} vs, siRNA_1^{METTL3}), ***$P = 0.0009$ (siRNA^{NTC} vs, siRNA_2^{METTL3}) and ***$P < 0.0001$ (siRNA^{NTC} vs, siRNA_P^{METTL3}). (**B**) Western Blot analysis of the protein expression levels of METTL3. Data represent the mean ± SEM. Data were analyzed using Student-T test. **$P = 0.0091$ (siRNA^{NTC} vs, siRNA_1^{METTL3}), *$P = 0.027$ (siRNA^{NTC} vs, siRNA_2^{METTL3}) and *$P = 0.028$ (siRNA^{NTC} vs, siRNA_P^{METTL3}). (**C**) Representative western blots showing expression of METTL3 and actin used as loading control. (**D**) Overall m^{6}A levels were measured using EpiQuik m^{6}A RNA Methylation Quantification Kit in ST*Hdh*$^{Q111/Q111}$ cells. Histograms show percentage of m^{6}A levels in total RNA ($n = 3$–4 independent experiments; 2 technical replicates/experiment). Data were analyzed using one-way ANOVA with Tukey´s multiple comparisons test. *$P = 0.0207$ (siRNA^{NTC} vs, siRNA_1^{METTL3}), ***$P = 0.0003$ (siRNA^{NTC} vs, siRNA_P^{METTL3}). (**E**) MazF-qPCR analysis of the DRACH motifs in *Htt* intron 1 ($n = 4$ independent experiments) in ST*Hdh*$^{Q111/Q111}$ transfected for 24 h with non-targeting control (NTC) and a pool of 3 target-specific siRNA (siRNA_P^{METTL3}) against METTL3. Data represent the mean ± SEM. Data were analyzed using Student-T test. **$P = 0.0043$ (siRNA^{NTC} vs, siRNA_P^{METTL3}). (**F**) qPCR analysis of the I1-pA1 *Htt* transcript in ST*Hdh*$^{Q111/Q111}$ transfected for 24h with non-targeting control (NTC), two different targeting sequences (siRNA_1^{METTL3} and siRNA_2^{METTL3}) and a pool of 3 target-specific siRNA (siRNA_P^{METTL3}) against METTL3 ($n = 4$ independent experiments). Data represent the mean ± SEM. Data were analyzed using one-way ANOVA with Tukey´s multiple comparisons test. *$P = 0.0309$, **$P = 0.0339$ compared with cells treated with siRNA^{NTC}. Source data are available online for this figure.
